# Supplementary material for: Targeting neddylation sensitizes colorectal cancer to topoisomerase I inhibitors by inactivating the DCAF13-CRL4 ubiquitin ligase complex
Source: Nat Commun. 2023 Jun 23;14:3762. doi: 10.1038/s41467-023-39374-9 (PMC10290057; doi:10.1038/s41467-023-39374-9)
Supplement: Supplementary file 1 — Supplementary Information [file 41467_2023_39374_MOESM1_ESM.pdf]

Supplementary Information

Supplementary Figure 1

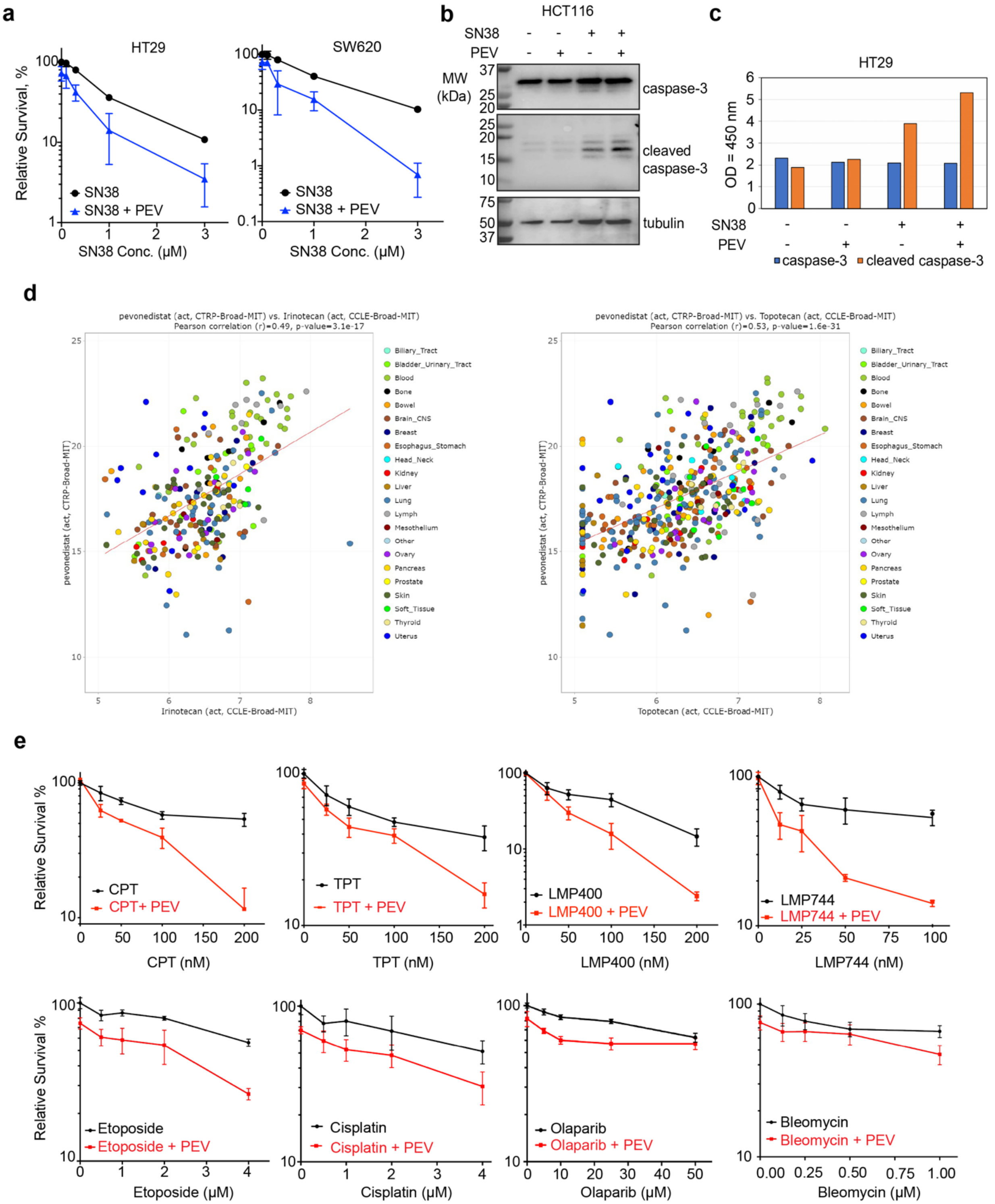

Supplementary Figure 1

a. Viability curves for 72 h treatments with SN38 in HT29 and SW620 cells (mean ± SD, N = 3 biologically independent experiments) using ATPLite luminescence Assay. 25 nM PEV was administered 4 h before SN38 exposure.

b. Western blotting in HCT116 cells assessing the induction of cleaved caspase-3 upon exposure to 10 µM SN38 for 6 h in the absence and presence of 10 µM PEV using indicated antibodies. N = 1.

c. ELISA in HT29 cells assessing the induction of cleaved caspase-3 upon exposure to 10 µM SN38 for 6 h in the absence and presence of 10 µM PEV.

d. Correlation plots of the activities of PEV and clinical TOP1 inhibitors irinotecan (left panel) and topotecan (right panel) across human cancer cell lines derived from CellMinerCDB. The statistic test was two-sided.

e. Viability curves for 72 h treatments with the indicated DNA damaging agents in HCT116 cells (mean ± SD, N = 3 biologically independent experiments) using ATPLite luminescence Assay. 25 nM PEV was administered 4 h before TOP1 inhibitor treatments including camptotecins camptotecin (CPT) and topotecan (TPT) and indenoisoquinolines LMP400 and LMP744. 50 nM PEV was administered 4 h before treatments with the other DNA damaging agents including topoisomerase II (TOP2) inhibitor etoposide, PARP inhibitor olarparib, alkylating agent cisplatin and DNA break inducer bleomycin.

Supplementary Figure 2

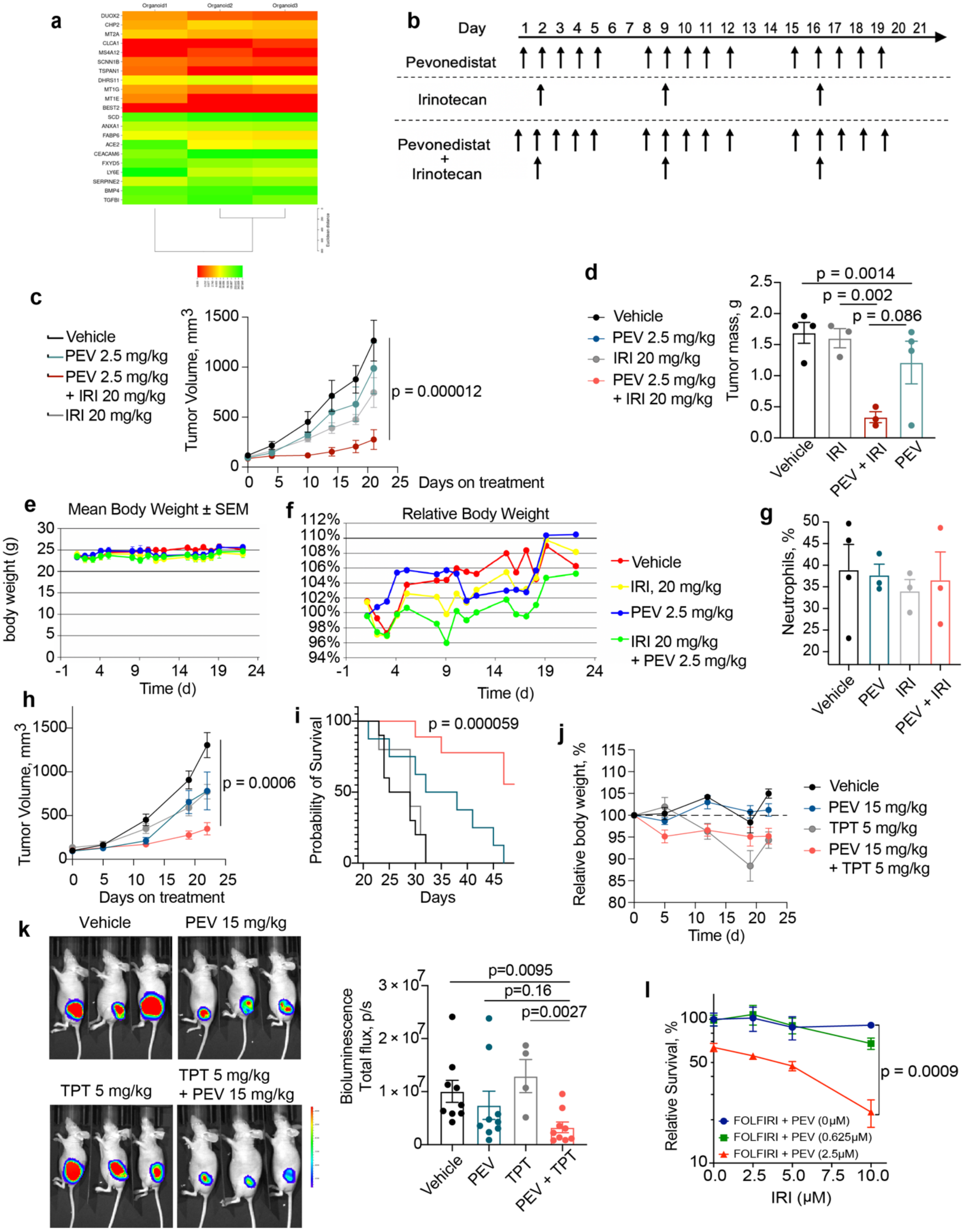

Supplementary Figure 2

- **a.** Hierarchical clustering by RNA-seq indicates differences in RNA expression profiling between the CRC patient-derived organoids.
- **b.** Experimental design schematics.
- **c.** Tumor growth in female athymic nude mice carrying subcutaneous HCT116 xenografts (n=10 per treatment group, except n=8 for PEV 2.5 mg/kg). p value determined by two-way ANOVA with multiple comparisons on day 21.
- **d.** Tumor weight measured in female athymic nude mice carrying subcutaneous HCT116 xenografts 48 h after the last dosing. p value determined by two-way ANOVA. Data are presented as mean ± SEM, n= 5 mice per treatment group.
- **e.** Mean body weight of the mice (n= 5 per treatment group).
- **f.** Body weight of the mice relative to day 1 of treatment (n= 5 per treatment group).
- **g.** Neutrophil counts in whole blood of the mice upon completion of the treatments on day 21. Data are presented as mean ± SEM, n= 3 mice per treatment group.
- **h.** Antitumor activity of a combination treatment of topotecan with PEV in female athymic nude mice carrying subcutaneous HCT116 xenografts (n=15 vehicle and combination group, n=14 PEV 15 mg/kg group, n=10 TPT 5 mg/kg). p value determined by two-way ANOVA with multiple comparisons on day 21.
- **i.** Kaplan-Meier survival curve of mice following the three treatment cycles (n=10/ group, except n=5 in TPT 5 mg/kg group). p value determined by Mantel-Cox test.
- **j.** Body weight relative to d1 of treatment (n=15 vehicle and combination group, n=14 PEV 15 mg/kg group, n=10 TPT 5 mg/kg).
- **k.** **Left panel:** representative bioluminescence images 21 days after treatment start for the combination treatment of topotecan with PEV; **Right panel:** Quantitation of bioluminescence imaging (n=9 per treatment group, except n=4 for TPT 5 mg/kg). Data are shown as mean ± SEM. p values determined by two-tailed Student’s t test.
- **l.** mCRC PDO #2 was treated with FOLFIRI (10 µM leucovorin, 800 µM fluorouracil and irinotecan hydrochloride of indicated concentrations) with PEV of indicated concentrations for 72 h for CellTiter-Glo Luminescent Cell Viability Assay (mean ± SD, N = 3 biologically independent experiments). The p-value was calculated using two-tailed student’s t-test.

Supplementary Figure 3

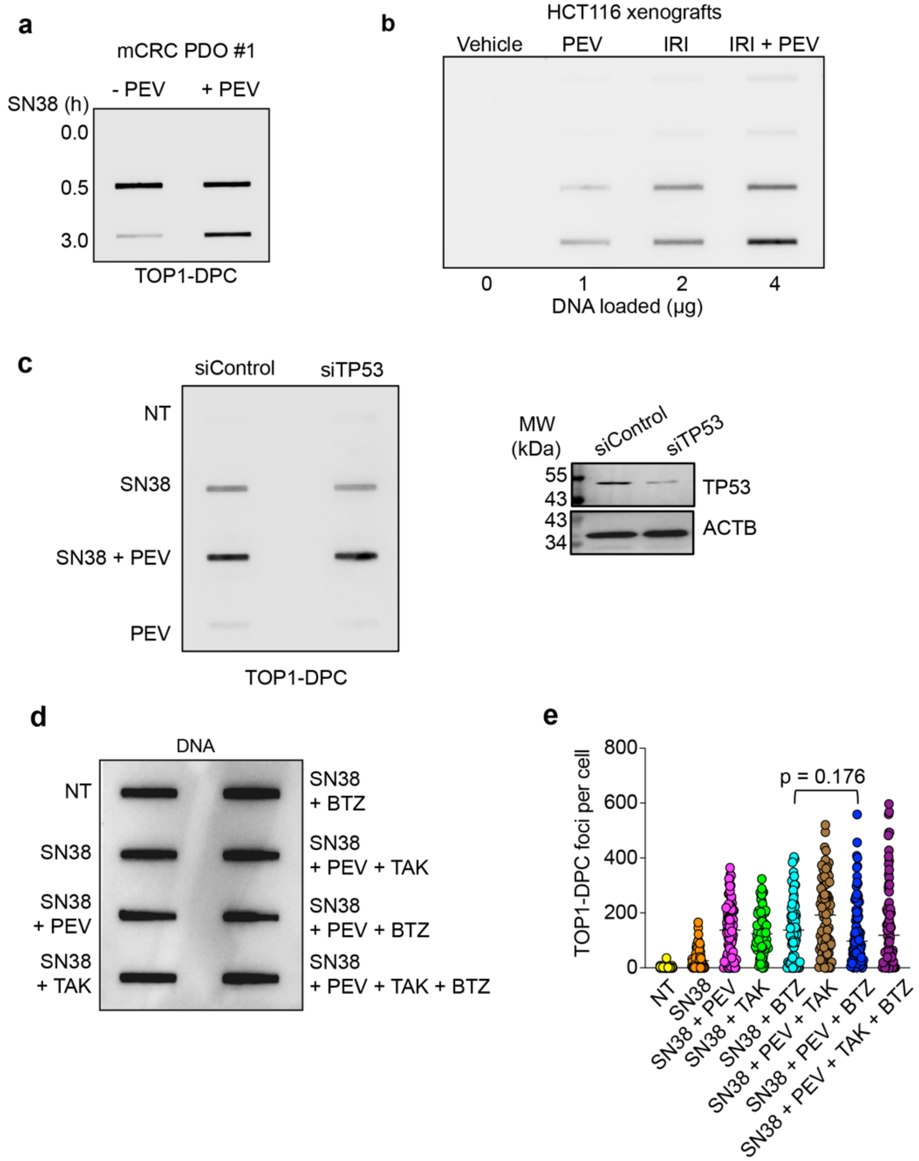

Supplementary Figure 3

a. mCRC PDO #1 treated with 1 μM SN38 in the absence or presence of PEV (10 μM, 1 h pre-treatment) and collected at indicated time points for ICE assay. TOP1-DPC was detected using anti-TOP1 antibody. N = 1.

b. HCT116 xenografts were treated with IRI 20 mg/kg and/or PEV 2.5 mg/kg and tumor tissues were collected by the end of the study for ICE assay. TOP1-DPC was detected using anti-TOP1 antibody. N = 1.

c. **Left panel:** HCT116 cells were transfected with control siRNA (siControl) or p53-targeting siRNA (siTP53) for 48 h then treated with 10 μM SN38 +/- 10 μM PEV for ICE assay. TOP1-DPC was detected using anti-TOP1 antibody. **Right panel:** WB confirming knocking down of p53 in HCT116 cells transfected with p53-targeting siRNA. N = 1.

d. ICE assay samples from Figure 3d were probed with anti-DNA antibody as loading control.

e. Quantitation of TOP1-DPC signals by immunofluorescence (IF) using anti-TOP1-DPC antibody in HCT116 cells treated with indicated drug combinations for 2 h: NT (no treatment), SN38 (1 μM), SN38 + PEV (10 μM, 1 h pretreatment), SN38 + TAK (10 μM, 1 h pretreatment), SN38 + BTZ (1 μM, 1 h pretreatment)**,** SN38 + PEV + TAK, SN38 + PEV + BTZ and SN38 + PEV + TAK + BTZ. Data are presented as mean ± SD, n = 706 total cells. The p-value was calculated using two-tailed student's t-test. Experiments were repeated 3 times.

Supplementary Figure 4

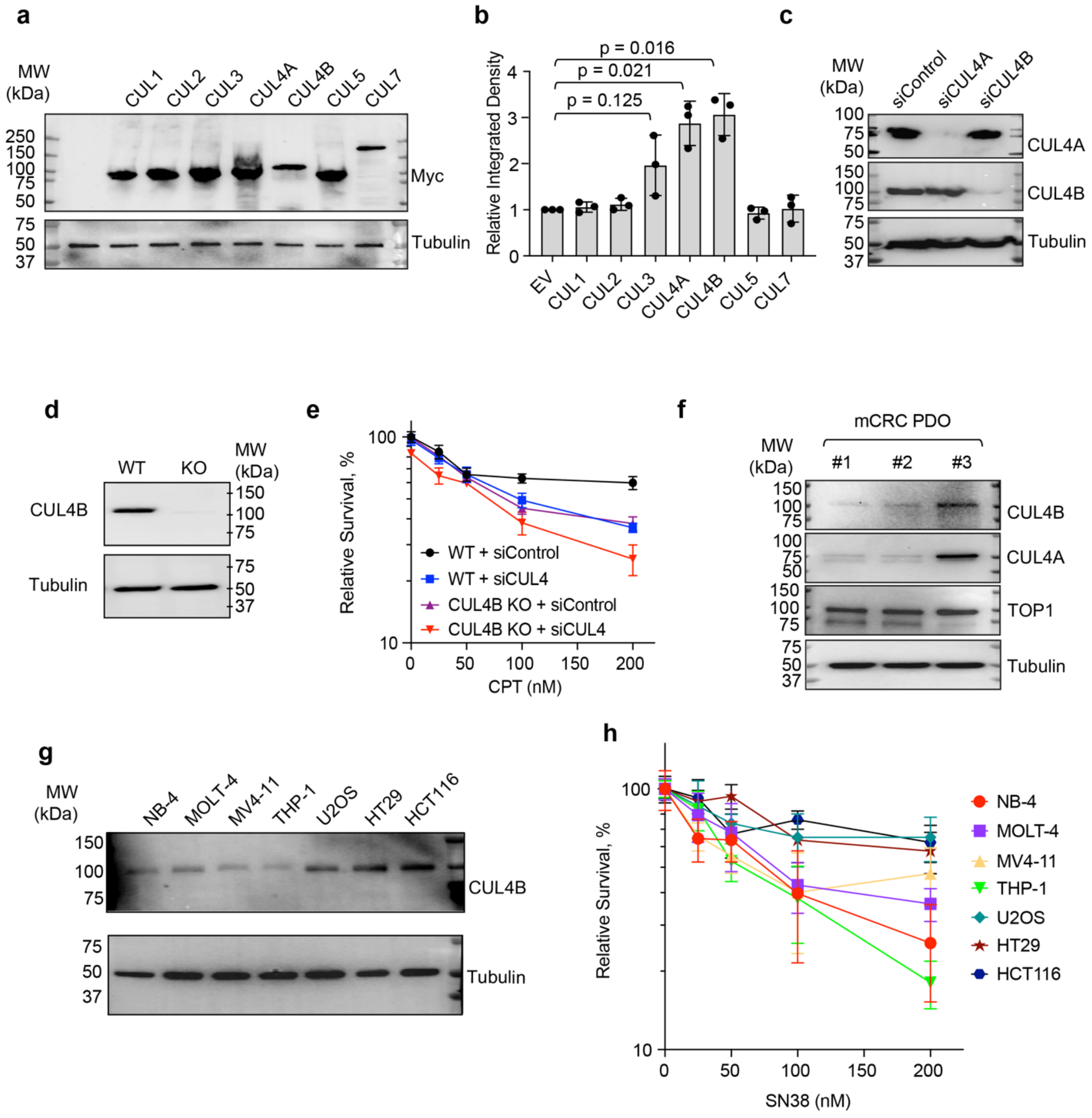

Supplementary Figure 4

a. Western blotting in HCT116 cells assessing the transfection efficiency of Myc-CUL1, Myc-CUL2, Myc-CUL3, Myc-CUL4A, Myc-CUL4B, Myc-CUL5 and Myc-CUL7 overexpression plasmids using anti-Myc antibody. N = 1.

b. Densitometric analyses of Ub-TOP1-DPCs from triplicate experiments including blots in Figure 4a. Density of Ub-TOP1-DPC/density of DNA of each group was normalized to that of cells transfected with EV only. Data are presented as mean values +/- SD, N = 3 biologically independent experiments. The p-value was calculated using two-tailed student's t-test.

c. Western blotting in HCT116 cells assessing the knockdown efficiency of CUL4A and CUL4B siRNAs using indicated antibodies. N = 1.

d. Western blotting in HCT116 cells confirming the CRISPR knockout of CUL4B using anti-CUL4B antibody. N = 1.

e. Viability curves for 72 h treatments with CPT of indicated concentrations in HCT116 WT and CUL4B KO cells transfected with or without CUL4A siRNA (mean ± SD, N = 3 biologically independent experiments).

f. WB in mCRC PDO #1, #2 and #3 assessing CUL4 and B proteins levels. N = 1.

g. WB in indicated cancer cell lines assessing CUL4 and B proteins levels. N = 1.

h. Viability curves for 72 h treatments with SN38 in indicated cancer cell lines (mean ± SD, N = 3 biologically independent experiments) using ATPLite luminescence Assay.

Supplementary Figure 5

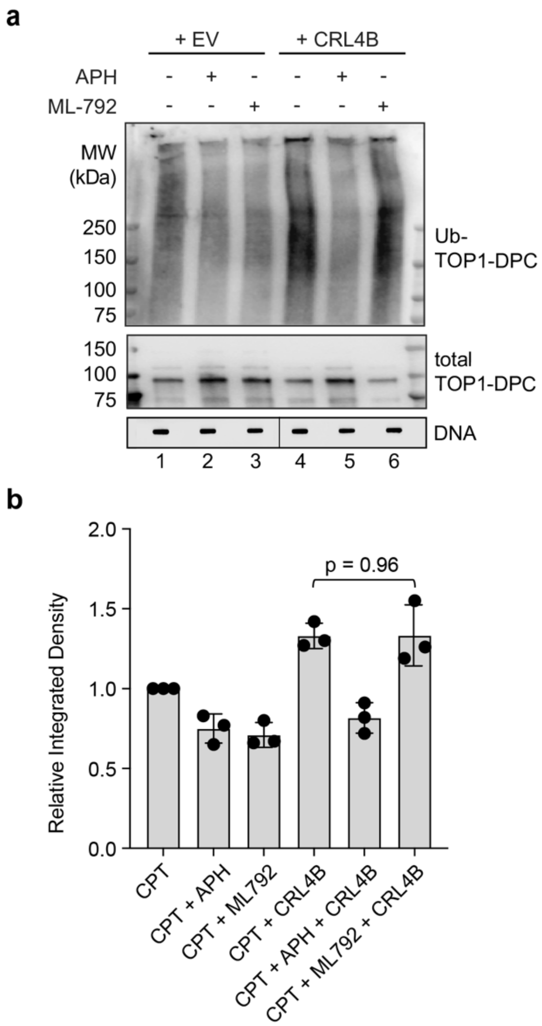

Supplementary Figure 5

a. HCT116 cells were transfected with empty vector (EV) or Myc-CUL4B + RBX1-FLAG overexpression plasmids (CRL4B) for 48 h before 2 h pre-treatment with replication inhibitor aphidicolin (APH, 10 μM) or SUMOylation inhibitor ML-792 (10 μM), followed by CPT treatment (20 μM, 30 min). The cells were then subjected to DUST assay for immunodetection of ubiquitylated TOP1-DPC and total TOP1-DPC using anti-ubiquitin and anti-TOP1 antibodies. The order of DNA slot blots has been altered, as indicated by the line, and that uncropped labelled blots can be found in the Source Data file.

b. Densitometric analyses of Ub-TOP1-DPCs from triplicate experiments including blots in Supplementary figure 5A. Density of Ub-TOP1-DPC/density of DNA of each group was normalized to that of cells transfected with EV only. Data are presented as mean values +/- SD, N = 3 biologically independent experiments.

Supplementary Figure 6

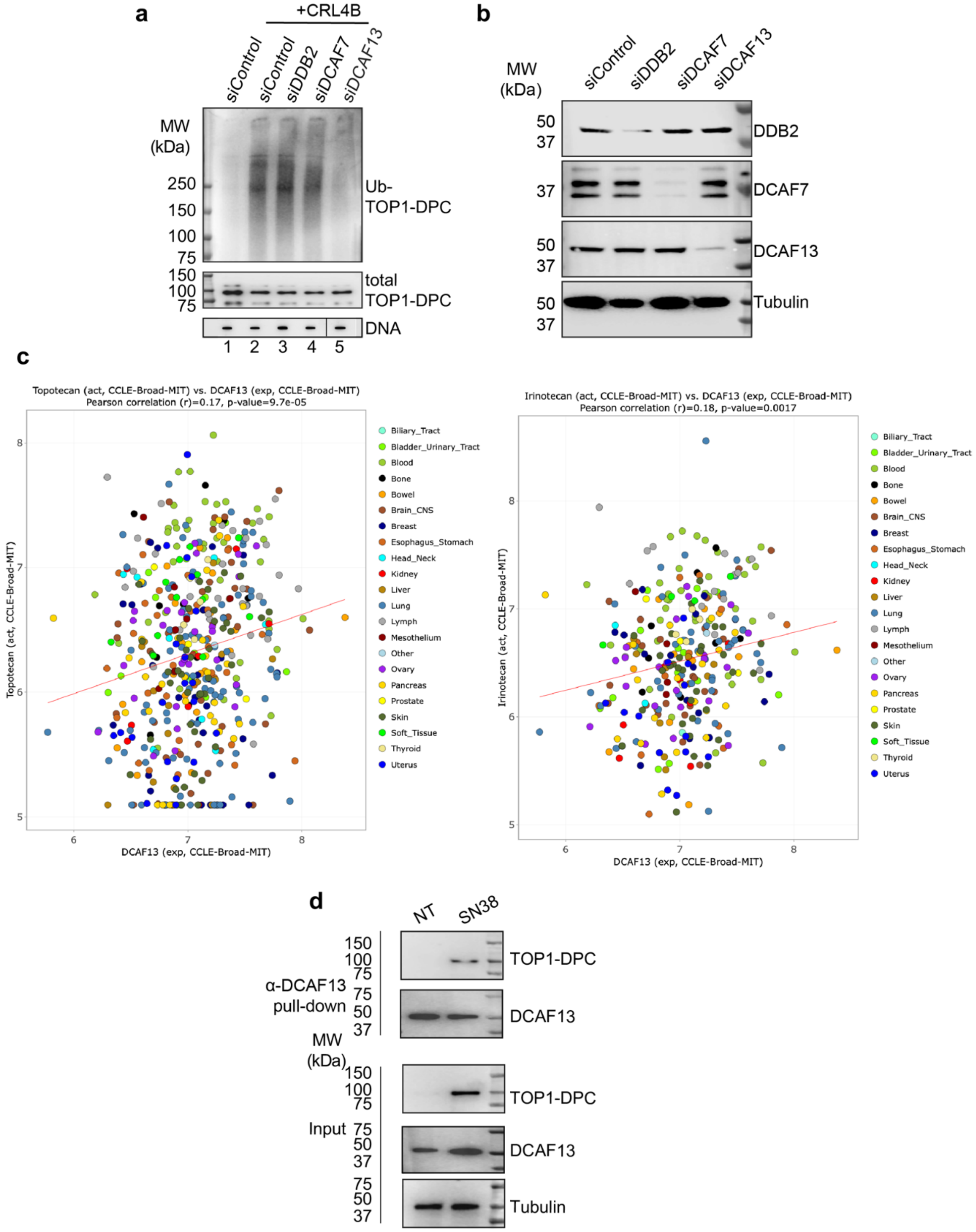

Supplementary Figure 6

- **a.** HCT116 cells were transfected with indicated siRNAs for 48 h, followed by CPT treatment (20 μM, 30 min) for DUST assay immunodetection of ubiquitylated total TOP1-DPCs using anti-ubiquitin and anti-TOP1 antibodies. N = 1. The order of DNA slot blots has been altered, as indicated by the line, and that uncropped labelled blots can be found in the Source Data file.
- **b.** Western blotting in HCT116 cells assessing the knockdown efficiency of DDB2, DCAF7 and DCAF13 siRNAs using indicated antibodies. N = 1.
- **c.** Correlation plots of the mRNA levels of DCAF13 and clinical TOP1 inhibitors topotecan (left panel) and irinotecan (right panel) across human cancer cell lines derived from CellMinerCDB.
- **d.** IP in HCT116 cells using anti-DCAF13 antibody was performed after 10 μM SN38 treatment for 30 min. IP samples and cell lysates (input) were subjected to immunoblotting (IB) with indicated antibodies. N = 1.

Supplementary Figure 7

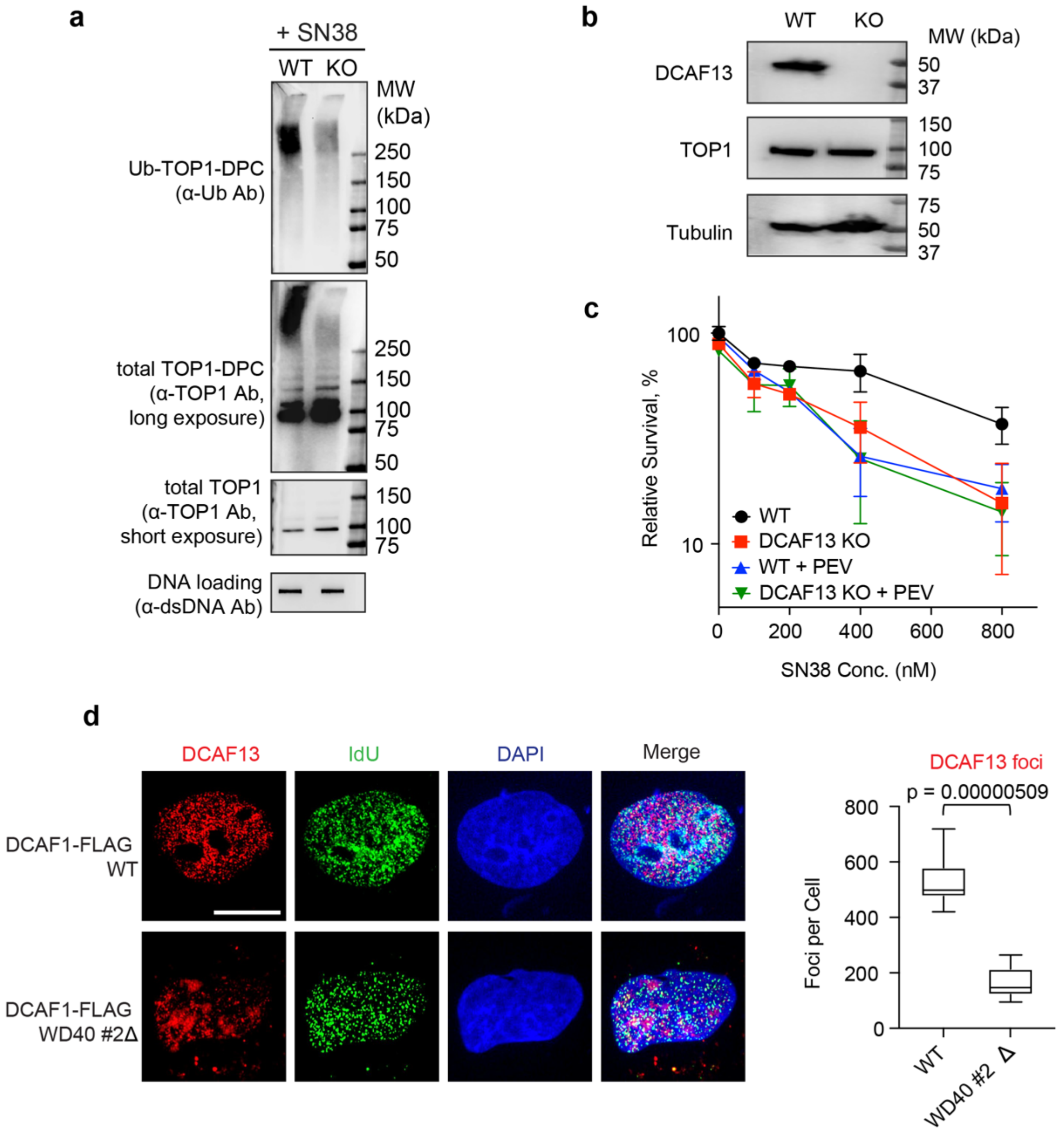

Supplementary Figure 7

a. DUST assay in HEK293 cells shows that CRISPR knocking-out of DCAF13 (KO) markedly decreased SN38-induced TOP1-DPC ubiquitylation (top panel) and increased total TOP1-DPC levels (middle panels) in comparison with the wild-type (WT) cells. 2 μg undigested DNA was loaded on slot-blot and probed with anti-dsDNA for the assay (bottom panel).

b. Western blotting in HEK293 cells confirming the CRISPR knockout of DCAF13. N = 1.

c. Viability curves for 72 h treatments with SN38 of indicated concentrations in HEK293 WT and DCAF13 KO cells (mean ± SD, n = 3 independent biological experiments).

d. **Left panel:** immunofluorescence in IdU-labeled HCT116 cells transfected with DCAF13-FLAG WT plasmid or DCAF13 WD40 repeat #2 Δ plasmid. Cells were pre-extracted and DCAF13 foci and IdU foci were monitored by instant structured illumination microscope (iSIM) using anti-FLAG and anti-BrdU antibodies. The scale bar represents 10 μm**. Right panel:** quantification of DCAF13 foci. The top is the maximum, the bottom is the minimum, and the middle line is the median. Whiskers are upper and lower quartiles. n = 20 total cells. The p-value was calculated using two-tailed student's t-test. Experiments were repeated 3 times.

Legends for the Supplementary Movie and Data files

Supplementary Movie 1
10-sec film of TOP1 HaloTag single-molecules in U2OS treated DMSO in HCT116 cells.

Supplementary Movie 2
10-sec film of TOP1 HaloTag single-molecules in U2OS after 2-h treatment of SN38 in HCT116 cells.

Supplementary Movie 3
10-sec film of TOP1 HaloTag single-molecules in U2OS after 2-h treatment of SN38 + PEV in HCT116 cells.

Supplementary Movie 4
10-sec film of TOP1 HaloTag single-molecules in U2OS after 2-h treatment of SN38 + TAK243 in HCT116 cells.

Supplementary Movie 5
10-sec film of TOP1 HaloTag single-molecules in U2OS after 2-h treatment of SN38 + BTZ in HCT116 cells.

Supplementary Movie 6
10-sec film of TOP1 HaloTag single-molecules in U2OS after 2-h treatment of SN38 + PEV + TAK243 in HCT116 cells.

Supplementary Movie 7
10-sec film of TOP1 HaloTag single-molecules in U2OS after 2-h treatment of SN38 + PEV + BTZ in HCT116 cells.

Supplementary Movie 8
10-sec film of TOP1 HaloTag single-molecules in U2OS after 2-h treatment of SN38 + PEV + TAK243 + BTZ in HCT116 cells

Supplementary Data 1
MIPE5.0 single-agent screen in HCT116 cells.

Supplementary Data 2
PEV Vs. all combination screen in HCT116 cells.

Supplementary Data 3
RNA-seq of CRC PDO #1, #2 and #3.

Supplementary Data 4
Toxicological studies of mice treated with irinotecan and PEV.

Supplementary Data 5
Proteomic analysis of His6-TOP1 interactome in HCT116 cells.

Uncropped blots in supplementary figures

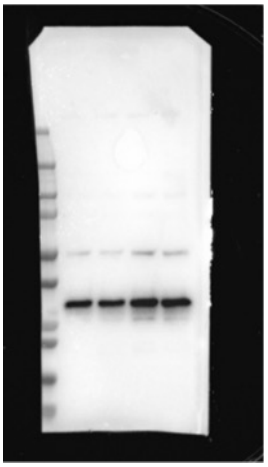

Supplementary Fig. 1b
caspase-3

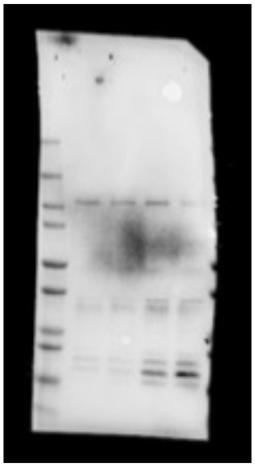

Supplementary Fig. 1b
cleaved caspase-3

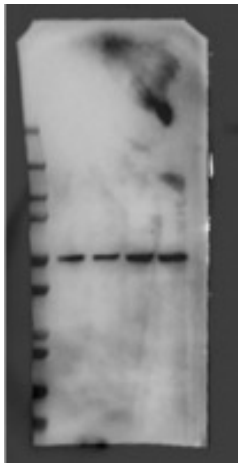

Supplementary Fig. 1b
tubulin

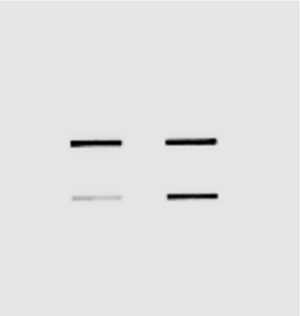

Supplementary Fig. 3a
TOP1-DPC

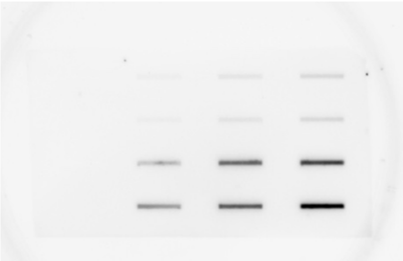

Supplementary Fig. 3b
TOP1-DPC

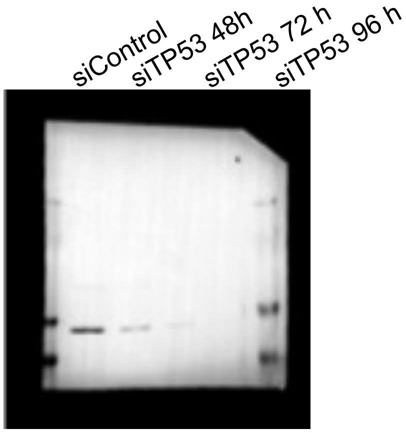

Supplementary Fig. 3c
TP53

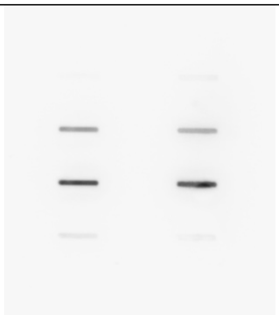

Supplementary Fig. 3c
TOP1-DPC

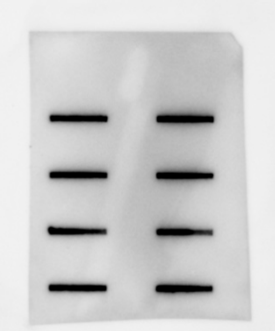

Supplementary Fig. 3d
DNA

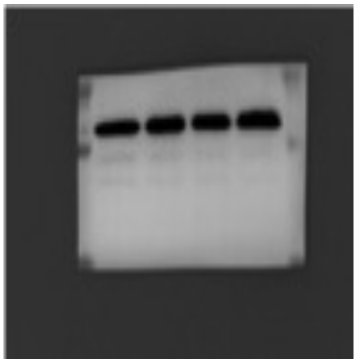

Supplementary Fig. 3c
tubulin

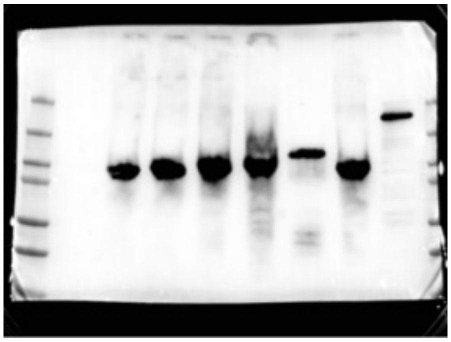

Supplementary Fig. 4a
myc

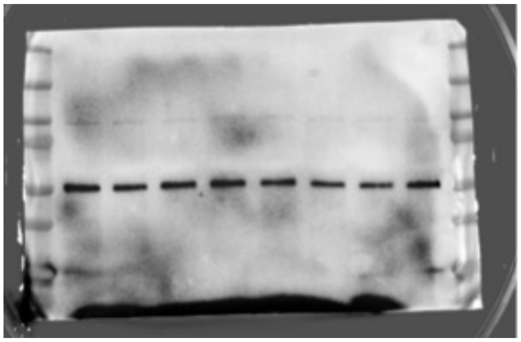

Supplementary Fig. 4a
tubulin

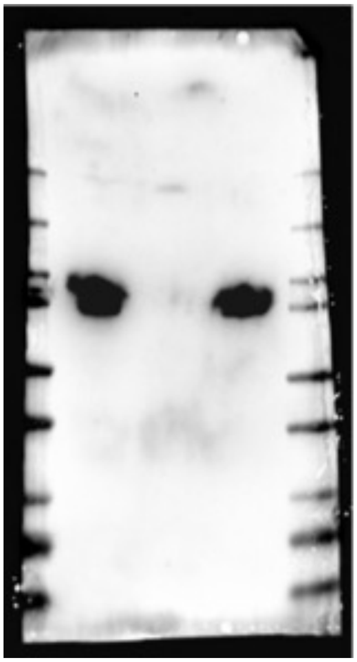

Supplementary Fig. 4a
CUL4A

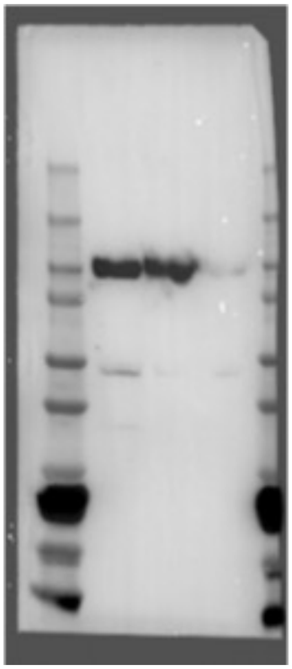

Supplementary Fig. 4a
CUL4B

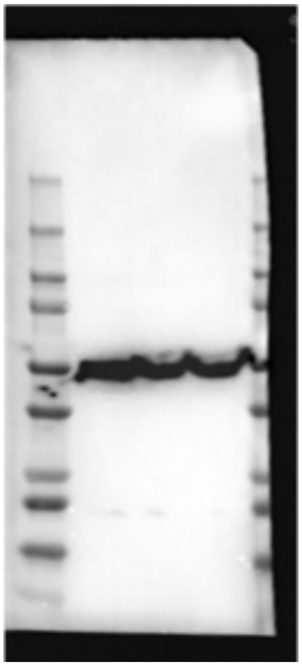

Supplementary Fig. 4a
tubulin

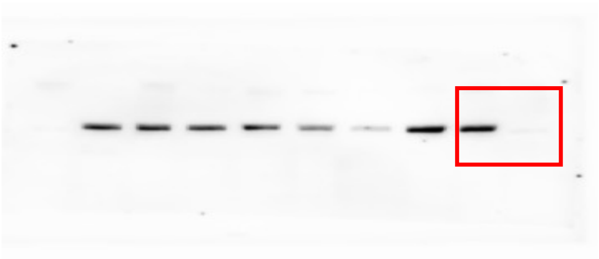

Supplementary Fig. 4d CUL4B

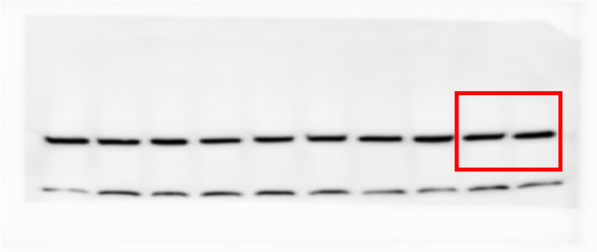

Supplementary Fig. 4d tubulin

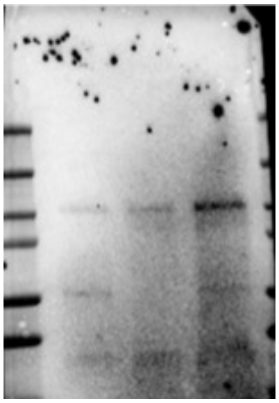

Supplementary Fig. 4f CUL4A

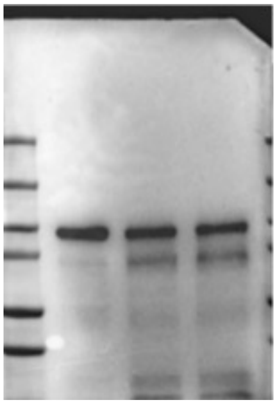

Supplementary Fig. 4f TOP1

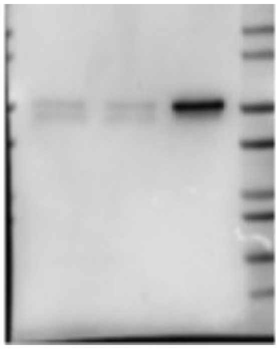

Supplementary Fig. 4f CUL4B

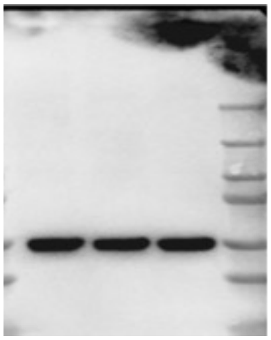

Supplementary Fig. 4f tubulin

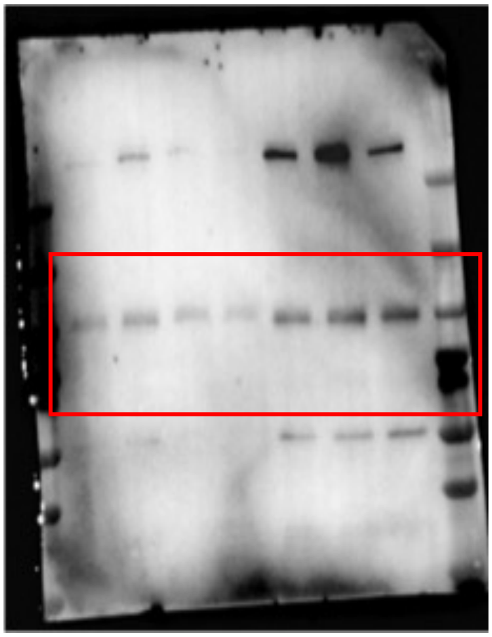

Supplementary Fig. 4g CUL4B

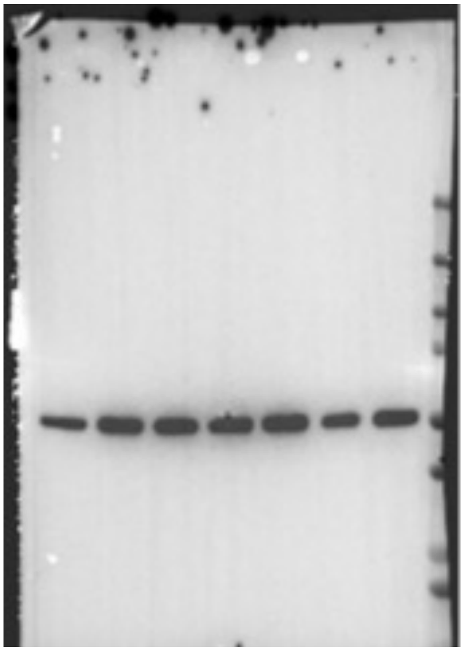

Supplementary Fig. 4g tubulin

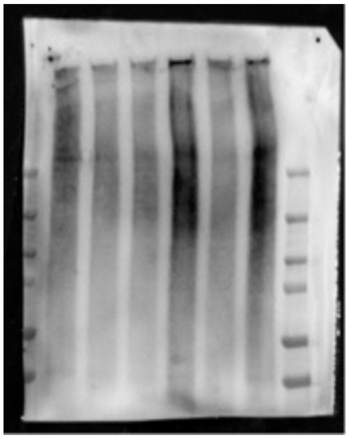

Replicate #1

Supplementary Fig. 5a
Ub-TOP1-DPC

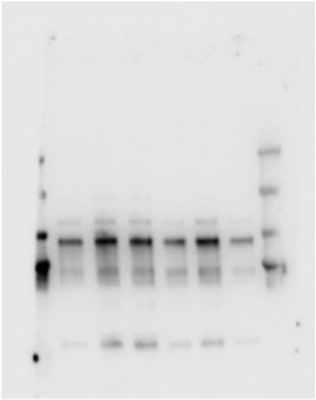

Replicate #1

Supplementary Fig. 5a
TOP1-DPC

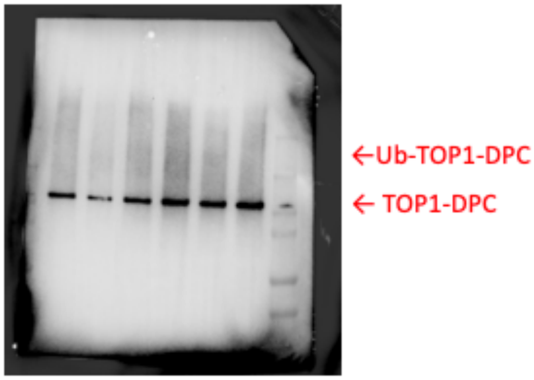

Replicate #2

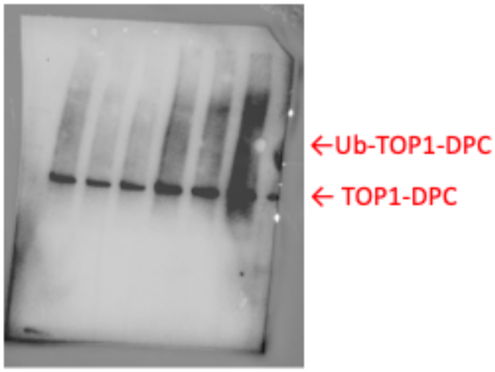

Replicate #3

Supplementary Fig. 5a Ub-TOP1-DPC and TOP1-DPC

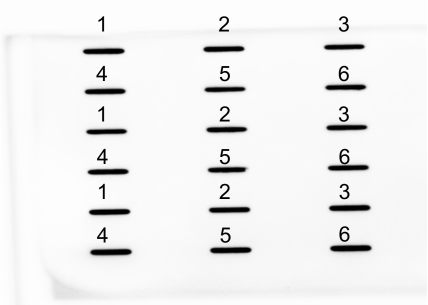

Replicate #1

Replicate #2

Replicate #3

Supplementary Fig. 5a DNA all three replicates

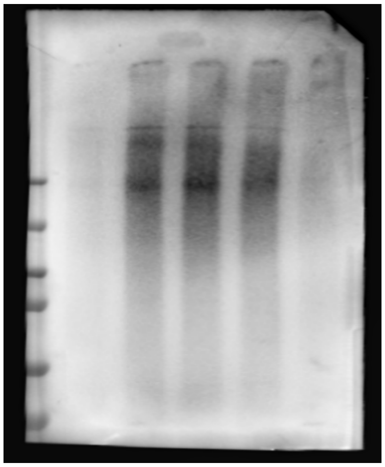

Supplementary Fig. 6a
Ub-TOP1-DPC

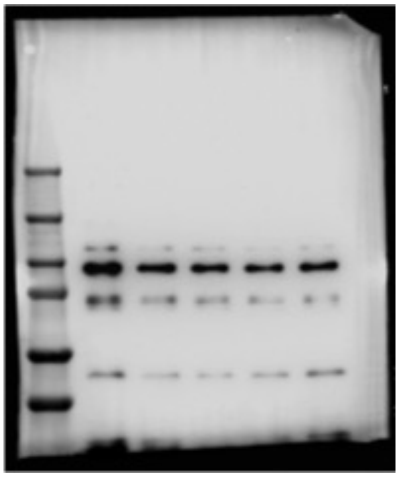

Supplementary Fig. 6a
TOP1-DPC

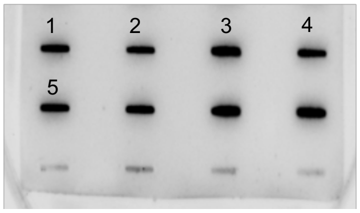

Supplementary Fig. 6a
DNA

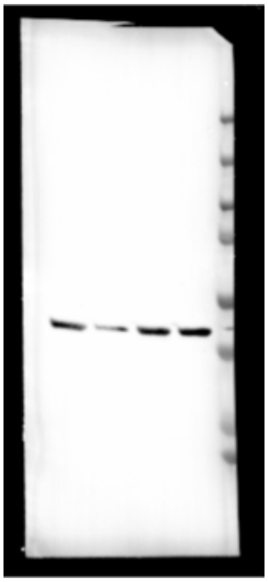

Supplementary Fig. 6b
DDB2

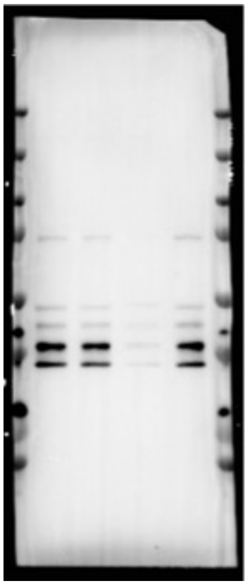

Supplementary Fig. 6b
DCAF7

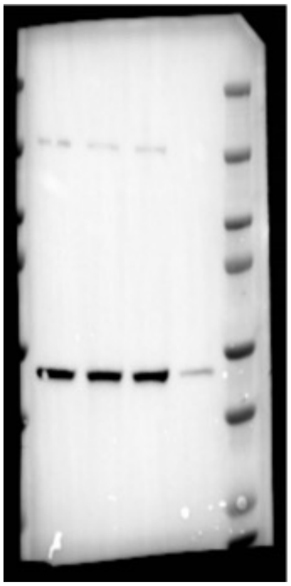

Supplementary Fig. 6b
DCAF13

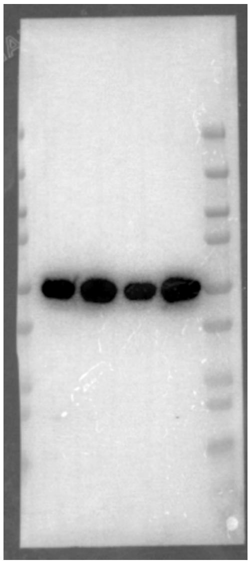

Supplementary Fig. 6b
Tubulin

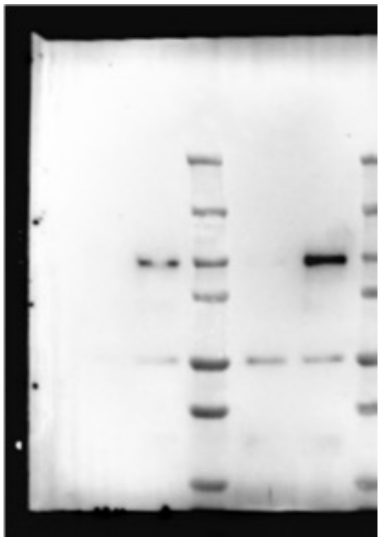

Supplementary Fig. 6d
TOP1-DPC IP and input

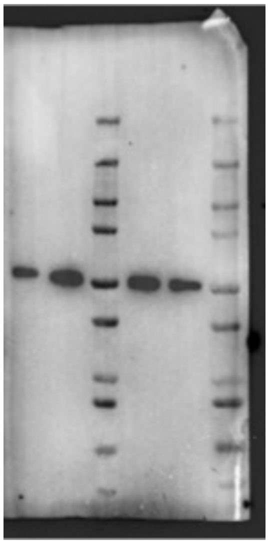

Supplementary Fig. 6d
DCAF13 input and IP

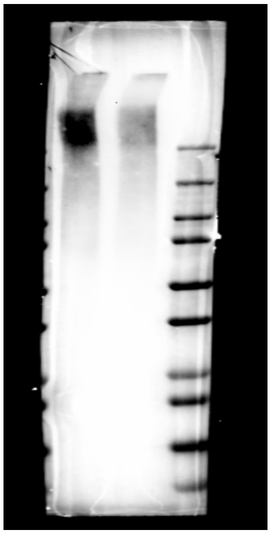

Supplementary Fig. 7a
Ub-TOP1-DPC
Replicate # 1

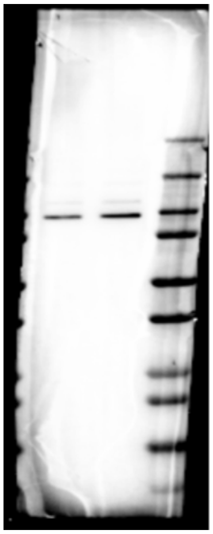

Supplementary Fig. 7a
TOP1-DPC
Replicate # 1

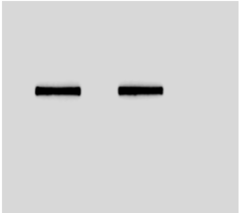

Supplementary Fig. 7a
DNA
Replicate # 1

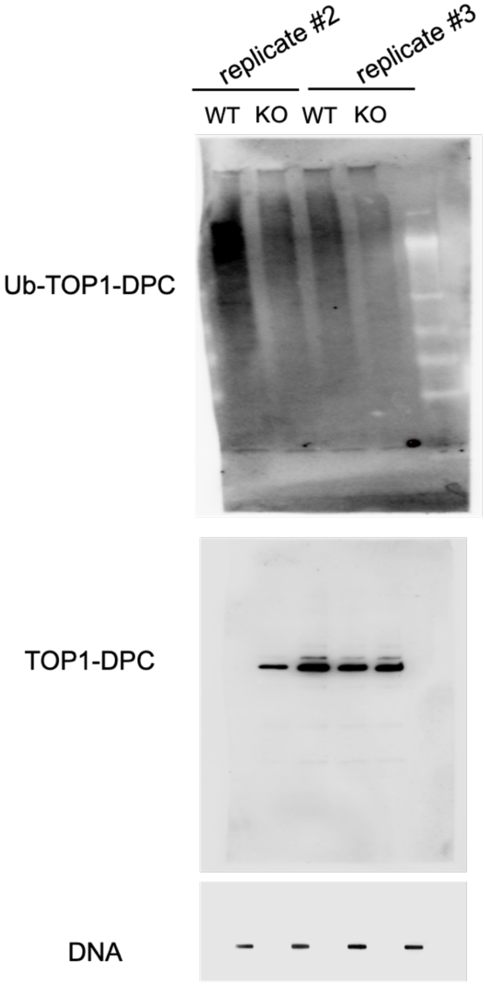

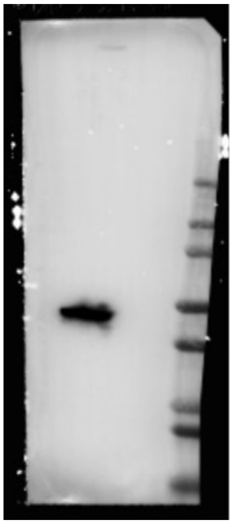

Supplementary Fig. 7b
DCAF13

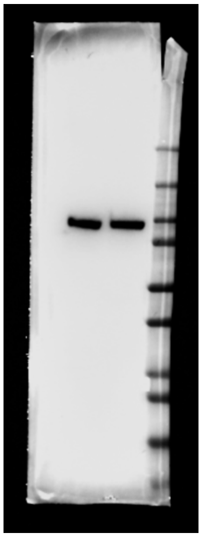

Supplementary Fig. 7b
TOP1

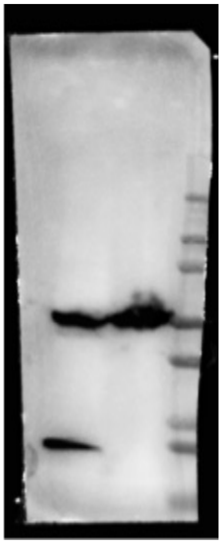

Supplementary Fig. 7b
tubulin
