# Supplementary material for: Targeting neddylation sensitizes colorectal cancer to topoisomerase I inhibitors by inactivating the DCAF13-CRL4 ubiquitin ligase complex
Source: Nat Commun. 2023 Jun 23;14:3762. doi: 10.1038/s41467-023-39374-9 (PMC10290057; doi:10.1038/s41467-023-39374-9)
Supplement: Supplementary file 2 — Description of Additional Supplementary Files [file 41467_2023_39374_MOESM2_ESM.pdf]

Title: Supplementary Movie 1
Description: 10-sec film of TOP1 HaloTag single-molecules in U2OS treated DMSO in HCT116 cells.

Title: Supplementary Movie 2
Description: 10-sec film of TOP1 HaloTag single-molecules in U2OS after 2-h treatment of SN38 in HCT116 cells.

Title: Supplementary Movie 3
Description: 10-sec film of TOP1 HaloTag single-molecules in U2OS after 2-h treatment of SN38 + PEV in HCT116 cells.

Title: Supplementary Movie 4
Description: 10-sec film of TOP1 HaloTag single-molecules in U2OS after 2-h treatment of SN38 + TAK243 in HCT116 cells.

Title: Supplementary Movie 5
Description: 10-sec film of TOP1 HaloTag single-molecules in U2OS after 2-h treatment of SN38 + BTZ in HCT116 cells.

Title: Supplementary Movie 6
Description: 10-sec film of TOP1 HaloTag single-molecules in U2OS after 2-h treatment of SN38 + PEV + TAK243 in HCT116 cells.

Title: Supplementary Movie 7
Description: 10-sec film of TOP1 HaloTag single-molecules in U2OS after 2-h treatment of SN38 + PEV + BTZ in HCT116 cells.

Title: Supplementary Movie 8
Description: 10-sec film of TOP1 HaloTag single-molecules in U2OS after 2-h treatment of SN38 + PEV + TAK243 + BTZ in HCT116 cells

Title: Supplementary Data 1
Description: MIPE5.0 single-agent screen in HCT116 cells.

Title: Supplementary Data 2
Description: PEV Vs. all combination screen in HCT116 cells.

Title: Supplementary Data 3
Description: RNA-seq of CRC PDO #1, #2 and #3.

Title: Supplementary Data 4
Description: Toxicological studies of mice treated with irinotecan and PEV.

Title: Supplementary Data 5
Description: Proteomic analysis of His6-TOP1 interactome in HCT116 cells.
