# Supplementary material for: Targeting neddylation sensitizes colorectal cancer to topoisomerase I inhibitors by inactivating the DCAF13-CRL4 ubiquitin ligase complex
Source: Nat Commun. 2023 Jun 23;14:3762. doi: 10.1038/s41467-023-39374-9 (PMC10290057; doi:10.1038/s41467-023-39374-9)
Supplement: Supplementary file 3 — Reporting Summary [file 41467_2023_39374_MOESM3_ESM.pdf]

nature portfolio

Corresponding author(s): Yilun Sun and Yves Pommier

Last updated by author(s): May 13, 2023

Reporting Summary

Nature Portfolio wishes to improve the reproducibility of the work that we publish. This form provides structure for consistency and transparency in reporting. For further information on Nature Portfolio policies, see our Editorial Policies and the Editorial Policy Checklist.

Statistics

For all statistical analyses, confirm that the following items are present in the figure legend, table legend, main text, or Methods section.

n/a	Confirmed	
☐	☒	The exact sample size (n) for each experimental group/condition, given as a discrete number and unit of measurement
☐	☒	A statement on whether measurements were taken from distinct samples or whether the same sample was measured repeatedly
☐	☒	The statistical test(s) used AND whether they are one- or two-sided<br>*Only common tests should be described solely by name; describe more complex techniques in the Methods section.*
☒	☐	A description of all covariates tested
☒	☐	A description of any assumptions or corrections, such as tests of normality and adjustment for multiple comparisons
☐	☒	A full description of the statistical parameters including central tendency (e.g. means) or other basic estimates (e.g. regression coefficient) AND variation (e.g. standard deviation) or associated estimates of uncertainty (e.g. confidence intervals)
☐	☒	For null hypothesis testing, the test statistic (e.g. F, t, r) with confidence intervals, effect sizes, degrees of freedom and P value noted<br>*Give P values as exact values whenever suitable.*
☒	☐	For Bayesian analysis, information on the choice of priors and Markov chain Monte Carlo settings
☒	☐	For hierarchical and complex designs, identification of the appropriate level for tests and full reporting of outcomes
☐	☒	Estimates of effect sizes (e.g. Cohen's d, Pearson's r), indicating how they were calculated

Our web collection on statistics for biologists contains articles on many of the points above.

Software and code

Policy information about availability of computer code

Data collection	Image Lab Software for PC Version 6.1
Data analysis	ImageJ 1.52a; Matlab R2020a; Prism 8.0; OriginPro 2019b

For manuscripts utilizing custom algorithms or software that are central to the research but not yet described in published literature, software must be made available to editors and reviewers. We strongly encourage code deposition in a community repository (e.g. GitHub). See the Nature Portfolio guidelines for submitting code & software for further information.

Data

Policy information about availability of data

All manuscripts must include a data availability statement. This statement should provide the following information, where applicable:

- Accession codes, unique identifiers, or web links for publicly available datasets
- A description of any restrictions on data availability
- For clinical datasets or third party data, please ensure that the statement adheres to our policy

Uncropped blots are provided in Source Data file, which has been deposited into figshare: https://doi.org/10.6084/m9.figshare.23408060.v1
RNA-seq data have been deposited into dbGaP under access number phs003257.v1.p1: https://www.ncbi.nlm.nih.gov/projects/gap/cgi-bin/study.cgi?study_id=phs003257.v1.p1. The RNA-seq dataset is under restricted access in a DAS for patient privacy laws. Please contact pommier@nih.gov to obtain the data without requiring approval from the DAC. Proteomic data have been deposited into MassIVE (MSV000091847,DOI:10.25345/C5RN30J25): https://

massive.ucsd.edu/ProteoSAFe/dataset.jsp?accession=MSV000091847.
All remaining data are available in the Article, Supplementary and Source Data files.

Human research participants

Policy information about studies involving human research participants and Sex and Gender in Research.

Reporting on sex and gender	male
Population characteristics	Caucasian
Recruitment	The qualifications for enrollment are a diagnosis of advanced cancer and potential eligibility for one of multiple cell therapy protocols. No selection bias.
Ethics oversight	NIH Institutional Review Board has approved the study with annual Protocol Reviews.

Note that full information on the approval of the study protocol must also be provided in the manuscript.

Field-specific reporting

Please select the one below that is the best fit for your research. If you are not sure, read the appropriate sections before making your selection.

[x] Life sciences [ ] Behavioural & social sciences [ ] Ecological, evolutionary & environmental sciences

For a reference copy of the document with all sections, see nature.com/documents/nr-reporting-summary-flat.pdf

Life sciences study design

All studies must disclose on these points even when the disclosure is negative.

Sample size	Sample size was determined to ensure statistical analyses. No statistical methods were used to predetermine sample size. For most of the experiments, 4-10 samples were used. Sample sizes were chosen based on a previous study PMID: 30622257.
Data exclusions	No data exclusions
Replication	ICE assays, His tag pulldown, cell and organoid survival assays and DUST assays were successfully repeated for 3 times. Single molecule imaging, confocal, PLA,<br>comet assays and iSIM microscopic analyses were successfully repeated two times.
Randomization	The experiments were randomized for the drug efficacy and toxicity studies in mice. Mouse allocation was random.
Blinding	The investigators were not blinded to allocation during experiments and outcome assessment. The investigate needed to design and control the experiments for treating certain mice, organoids andcells with certain drugs for the study. The investigators had to know what mice, organoids and cell lines and what drugs they are dealing with.

Reporting for specific materials, systems and methods

We require information from authors about some types of materials, experimental systems and methods used in many studies. Here, indicate whether each material, system or method listed is relevant to your study. If you are not sure if a list item applies to your research, read the appropriate section before selecting a response.

Materials & experimental systems

n/a	Involved in the study	
[ ]	[x]	Antibodies
[ ]	[x]	Eukaryotic cell lines
[x]	[ ]	Palaeontology and archaeology
[ ]	[x]	Animals and other organisms
[x]	[ ]	Clinical data
[x]	[ ]	Dual use research of concern

Methods

n/a	Involved in the study	
[x]	[ ]	ChIP-seq
[x]	[ ]	Flow cytometry
[x]	[ ]	MRI-based neuroimaging

Antibodies

Antibodies used	Anti-α Tubulin, rat polyclonal, Santa Cruz, Cat# sc-53030; Anti-ubiquitin, mouse monoclonal (P4D1), Santa Cruz, Cat# sc-8017; Anti-phospho-Histone H2A.X (Ser139), mouse monoclonal, Millipore, Cat# 05-636-I; Anti-pRPA32 (Ser4/Ser8), rabbit polyclonal, Bethyl Lab, Cat# A300-245A; Anti-pCHK1 (Ser345), rabbit monoclonal, Cell Signaling, Cat# 2348; Anti-TOP1, mouse monoclonal (C21), BD Biosciences, Cat# 556597; Anti-TOP1-DPC, mouse monoclonal (1.1A), Millipore, Cat# MABE1084; Anti-His tag, rabbit monoclonal, Cell Signaling, Cat# 12698; Anti-FLAG, mouse monoclonal (M2), Sigma, Cat# F1804; Anti-FLAG, rabbit polyclonal, Sigma, Cat# F7425; Anti-Myc, mouse monoclonal (9B11), Cell Signaling, Cat# 2276; Anti-dsDNA, mouse monoclonal (35I9), Abcam, Cat# ab27156; Anti-CUL4A, rabbit polyclonal, Bethyl Lab, Cat# A300-739A; Anti-CUL4B, Novus Biological, rabbit polyclonal, Cat# H00008450-B01P; Anti-DDB2, rabbit polyclonal, Thermo Fisher, Cat# PA5-37361; Anti-DCAF7, rabbit polyclonal, Thermo Fisher, Cat# PA5-93222; Anti-DCAF13, rabbit monoclonal, Abcam, Cat# ab195121; Anti-p53, rabbit monoclonal, Cell Signaling Technology, Cat# 9282. Anti-cleaved caspase-3, rabbit polyclonal, Abcam, ab2302. Goat anti-mouse IgG (H+L) Cross-Adsorbed Secondary Antibody, Thermo Fisher, A-11001. Alexa Fluor™ 488 Goat anti-rabbit IgG (H+L) Cross-Adsorbed Secondary Antibody, Alexa Fluor™ 568, Thermo Fisher, A-11011. All the antibodies were used at 1: 1000 dilution.
Validation	All the used antibodies have been validated by Western blotting and or immunofluorescence in human cells by the vendors. Validation statements are available on the manufacturer's websites.

Eukaryotic cell lines

Policy information about cell lines and Sex and Gender in Research

Cell line source(s)	HCT116 colorectal cancer cells, HT29 colorectal cancer cells, HEK293 human embryo kidney cells, HCT15, KM12, SW837, SW48 and SW620 colorectal cancer cells were obtained from ATCC.
Authentication	Cell Line Authentication was carried out using Short Tandem Repeat Analysis at Frederick National Laboratory.
Mycoplasma contamination	Cells were routinely tested for mycoplasma by MycoAlert (Lonza) and found negative.
Commonly misidentified lines (See ICLAC register)	No commonly misidentified cell line was used.

Animals and other research organisms

Policy information about studies involving animals; ARRIVE guidelines recommended for reporting animal research, and Sex and Gender in Research

Laboratory animals	8 weeks female athymic nude mice were obtained from the NCI-Frederick Mouse Repository (MD, USA).
Wild animals	No wild animals were used in this study
Reporting on sex	Female. Sex was not considered in the study design and analysis.
Field-collected samples	No field-collected animals were used in this study
Ethics oversight	All procedures were performed in compliance with protocols approved by the NIH Institutional Animal Care and Use Committee and were in accordance with federal guidelines for the humane treatment and care of laboratory animals. The study protocol was approved by the NCI Animal Care and Use Committee

Note that full information on the approval of the study protocol must also be provided in the manuscript.
